# Healthcare worker knowledge and skills following coaching in WHO early essential newborn care program in the Solomon Islands: a prospective multi-site cohort study

**DOI:** 10.1186/s12884-020-2739-z

**Published:** 2020-02-07

**Authors:** Shidan Tosif, Anna Jatobatu, Anita Maepioh, Amy Gray, Howard Sobel, Priya Mannava, Trevor Duke

**Affiliations:** 1 Centre for International Child Health, University of Melbourne, Murdoch Children’s Research Institute, Royal Children’s Hospital Melbourne, 50 Flemington Rd, Parkville, VIC 3052 Australia; 2 National Newborn Health Coordinator, Reproductive and Child Health Department, Ministry of Health and Medical Services, Honiara, Solomon Islands; 3 Department of Obstetrics and Gynaecology, National Referral Hospital, Honiara, Solomon Islands; 4 Coordinator Maternal, Child Health and Quality and Safety, World Health Organization Regional Office for the Western Pacific, Manila, Philippines; 5 Technical Officer, Surveillance, Information and Accountability, Reproductive, Maternal, Newborn, Child and Adolescent Health, World Health Organisation Regional Office for the Western Pacific, Manila, Philippines

**Keywords:** Neonatal resuscitation, Neonatal training programs, Low- and middle-income countries, Small Island developing states

## Abstract

**Background:**

Newborn mortality in Oceania declined slower than other regions in the past 25 years. The World Health Organization (WHO) introduced the Early Essential Newborn Care program (EENC) in 2015 in Solomon Islands, a Small Island Developing State, to address high newborn mortality. We explored knowledge and skills retention among healthcare workers following EENC coaching.

**Methods:**

Between March 2015 and December 2017, healthcare workers in five hospitals were assessed: pre- and post-clinical coaching and at a later evaluation. Standardised written and clinical skills assessments for breathing and non-breathing baby scenarios were used. Additionally, written surveys were completed during evaluation for feedback on the EENC experience.

**Results:**

Fifty-three healthcare workers were included in the evaluation. Median time between initial coaching and evaluation was 21 months (IQR 18–26). Median written score increased from 44% at baseline to 89% post-coaching (*p* < 0.001), and was 61% at evaluation (*p* < 0.001). Skills assessment score was 20% at baseline and 95% post-coaching in the Breathing Baby scenario (*p* < 0.001). In the Non-Breathing Baby scenario, score was 63% at baseline and 86% post-coaching (*p* < 0.001). At evaluation, median score in the Breathing Baby scenario was 82% a reduction of 13% from post-coaching (*p* < 0.001) and 72% for the Non-Breathing Baby, a reduction of 14% post-coaching (*p* < 0.001). Nurse aides had least reduction in evaluation scores of − 2% for the Breathing Baby and midwives − 10% for the Non-Breathing Baby respectively from post-coaching to evaluation.

**Conclusions:**

EENC coaching resulted in immediate improvements in knowledge and skills but declined over time. Healthcare workers who used the skills in regular practice had higher scores. Complementary quality improvement strategies are needed to sustain resuscitation skills following training over time.

**Trial registration:**

Australia New Zealand Trial Registry, Retrospective Registration (12/2/2019), registration number ACTRN12619000201178.

## Background

The Oceania region (excluding Australia and New Zealand) experienced the slowest rate of reduction in neonatal mortality compared to other developing regions during the period of the Millennium Development Goals [1]. Like other lower-middle income Small Island Developing States (SIDS) [2], the small health workforce in the Solomon Islands faces enormous accessibility issues including dispersed populations, challenging geographies and environmental vulnerability to natural disasters.

Many gaps exist in the health facility care of common causes of neonatal mortality: intrapartum-related complications, complications of prematurity and severe infection [3, 4]. Neonatal resuscitation training in facilities can reduce early deaths [3, 5, 6]. Supportive interventions in the immediate newborn period such as skin-to-skin contact, early and exclusive breastfeeding and temperature control have been shown to improve outcomes [7]. A combined approach including early essential newborn care, resuscitation, and basic care of common neonatal problems could address the most common causes of perinatal mortality [8].

Continuing professional development for health care workers in Solomon Islands presents challenges due to few opportunities to learn or refresh knowledge and skills, and this is a barrier to improving newborn quality of care. The World Health Organisation (WHO) Early Essential Newborn Care (EENC) program focuses on developing skills and knowledge for the management of “Breathing” and “Non-breathing” babies. It addresses the critical first moments and days of newborn care for healthcare workers in health facilities, where over 95% of births take place in the Western Pacific [9–11]. Key content areas are neonatal resuscitation and basic newborn care, with emphasis on thorough drying, delayed cord clamping, skin-to-skin contact, reduction of harmful practices and support for exclusive breastfeeding. Whilst these measures do not require sophisticated technology, they rely on healthcare worker skills and knowledge to reduce risk of infection or death around the time of birth [4].

The aim of this study was to determine the impact on skills and knowledge amongst healthcare workers following EENC training, and characterise the healthcare provider attributes that effect retention and practice in the unique healthcare context of Solomon Islands.

## Methods

### Description of intervention

EENC was implemented as the country-level program for newborn care in Solomon Islands. The Ministry of Health and Medical Services (MHMS) rolled out the program across the nine provinces in the country from 2015 onwards. Following a national training, a newborn nurse coordinator (AJ) delivered subsequent trainings at the National Referral Hospital (NRH) and provincial hospitals with 1–2 nurse or midwife co-facilitators. The 2-day EENC program consisted of on-site coaching, with a low participant to facilitator ratio (6:1) [11]. The venue for training was the delivery room, or, where not possible, a room setup with a similar arrangement. The coaching approach was participatory in style without didactic teaching. In small groups, a participant would first demonstrate normal practice. The facilitators then explored why certain actions were taken, with discussion of the evidence for correct practice, as well as the evidence that some practices are unnecessary or potentially harmful (e.g. unnecessary routine suctioning, early washing the baby, separation of the baby and the mother). Participants were invited to share feedback in a supportive way, pointing out correct actions or if improvement was required. Each participant then took part in repeated practice whilst the facilitator used the EENC skills checklist as a reference. At the end of the coaching, the establishment of a quality improvement team within 3 months was planned at each site, with relevant guidance provided from EENC modules.

### Setting

Solomon Islands shares many geographical and demographic characteristics with other low- and middle-income countries in Oceania and SIDS globally. A population of almost 600,000 is dispersed amongst nine provinces and more than 900 islands. For this study, the Solomon Islands MHMS purposively selected five hospitals which together serve 80% of the national population: the NRH, Gizo Hospital, Kil’ufi Hospital, Makira Hospital, and Good Samaritan hospitals.

### Study design and participants

We conducted a pre and post, multiple-site, facility-based study between March 2015 and November 2017. Eligible participants were health care workers who attended EENC coaching and were evaluated prior to refresher training.

**Fig. 1:**
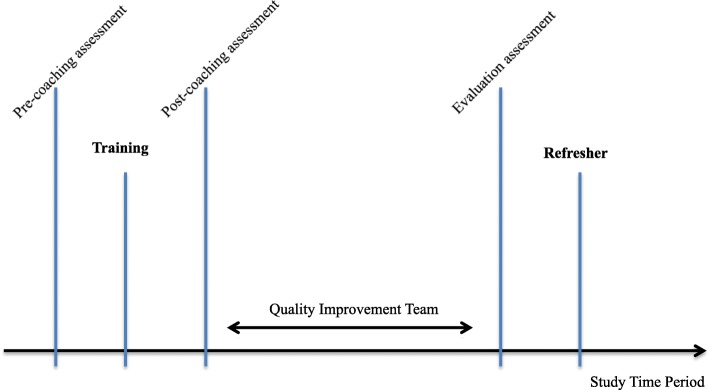
Sequence of study period activities

The primary outcome measures were knowledge and simulated skill scores according to standardised assessments contained in EENC, as have been used in the implementation of EENC throughout the Western Pacific region [12] and are available online [11]. Baseline scores for knowledge and skills were established from pre-coaching assessment of one random participant in each group. Assessment of all participants occurred immediately following EENC coaching, and prior to a refresher (see Fig. 1 for timeline). Timing of refresher was aimed for 12-months post-coaching.

Knowledge was assessed with a written test, with questions on the themes of routine newborn care, breast-feeding and resuscitation. The skills assessment tested skills in managing a mother and newborn in two scenarios, the delivery of a “Breathing” and “Non-breathing” baby. The written test and scenarios were carried out in a room set-up for the purpose under examination conditions.

Demographic data was collected using a confidential written questionnaire including experience, qualifications, work location, and attendance at other neonatal training (see Additional file 1). We asked participants about their satisfaction of the content of EENC using a 5-point Likert scale (1 = Too complicated, 2 = A little complicated, 3 = Just right, 4 = A little simple, 5 = Too simple). Opportunity was given to clarify questions using Solomon Islands Pigin during the written component and skills assessment scenarios were carried out in Solomon Islands Pigin or English according to participant preferences. The assessment team consisted of midwife (AM), newborn nurse coordinator (AJ) and visiting paediatrician (ST).

### Analysis

Data were summarised with frequencies or percentages for categorical values, or means and standard deviations (SD) or medians and interquartile ranges (IQR) for continuous variables. Difference testing between pre-coaching and post-coaching groups was performed using Mann-Whitney U test. Comparison between pairs of scores from health care workers between post-coaching to refresher was performed using the Paired Sign Test. Testing for differences of continuous variables between groups was performed using the Kruskal-Wallis test.

Written and skill assessment scores were manually entered from data collection forms into Excel, before cleaning and analysis in STATA (Version 15.0). This study was approved by the Solomon Islands Health Research and Ethics Review Board (project number HRE033/16) and the University of Melbourne Human Research Ethics Committee (HREC number 1646267.1). This study adheres to STROBE guidelines for reporting observational studies [13].

## Results

A total of 53 participants were included. Pre-coaching baseline scores were established from 25 participants in the Breathing Baby scenario, and 15 participants in the Non-Breathing Baby scenario. Median time for participants between coaching and evaluation was 21 months (IQR 18–26). There were 23 nurses, 15 doctors, 8 midwives and 7 nurse aides. The average healthcare worker was 36 years of age (±12 years), and had 9 years experience in neonatal care (±7 years). Six-per cent (3/53) of healthcare workers participated in a quality improvement activity following training. Thirty-one per cent (16/53) of healthcare workers had worked in two or more health facilities in the last 5 years. Forty-seven per cent (25/53) of healthcare workers had past training related to newborn care, none had received a refresher or follow up training previously. Past trainings was in Integrated Management of Childhood Illnesses, World Health Organisation Hospital Care for Children, Mother Baby Friendly Hospital Initiative, University based or other visiting programmatic trainings in newborn care.

### Evaluation of written scores

**Fig. 2:**
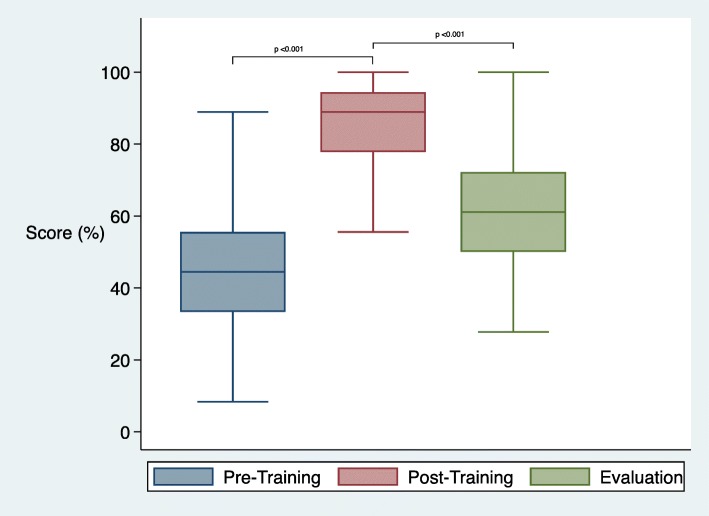
Written score assessment results by time period

All healthcare workers participated in the written test (*n* = 53) (Fig. 2). There was a significant increase in written scores immediately following coaching. Median written scores increased 45% from 44% (IQR 33–56) to 89% (IQR 78–94) (*p* < 0.001). At the time of the evaluation, median scores were 61%, (IQR 50–72) a reduction of 28% (*p* < 0.001) from post-coaching levels but still 17% higher than at baseline.

### Evaluation of skills

**Fig. 3:**
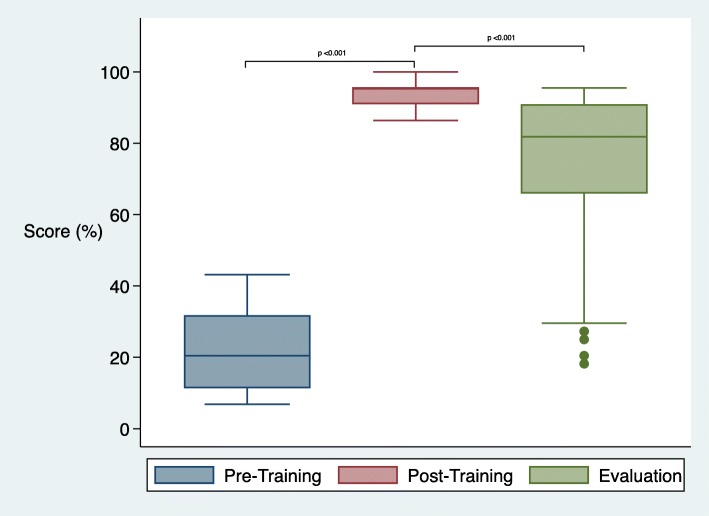
Skill scores in breathing baby scenario by time period

**Fig. 4:**
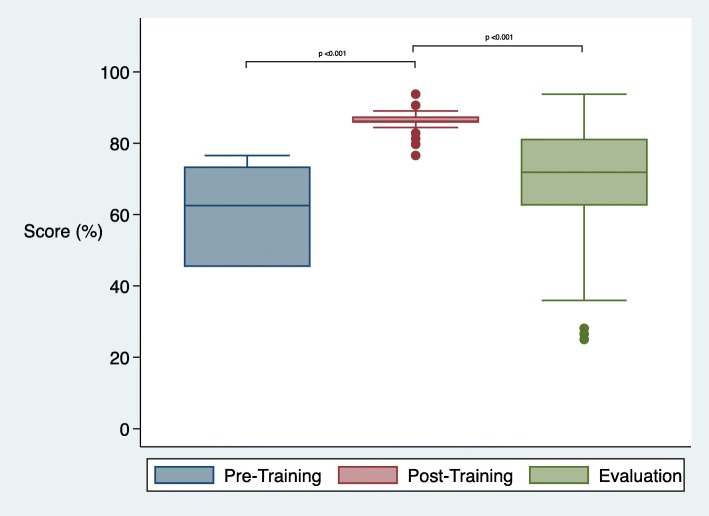
Skill scores in non-breathing baby by time period

Median scores in the Breathing Baby scenario went from 20% (IQR 11–32) pre-coaching, to 95% (IQR 91–95) post-coaching, and 82% (IQR 66–91) at time of evaluation (*p* < 0.001) (Fig. 3). Median scores in the Non-Breathing Baby scenario were 63% (IQR 45–73) pre-coaching, 86% (IQR 86–88) post-coaching and 72% (IQR 63–81) at evaluation (*p* < 0.001) (Fig. 4). In the Breathing Baby scenario, health care workers who received past training did not have better retention than those who had not received training (− 20% and − 14% respectively, *p* = 0.291), and had poorer performance in the Non-Breathing Baby scenario (− 13% vs − 22%, *p* = 0.040).

In the Non-Breathing Baby scenario, mean scores for neonatal resuscitation skills were lower than routine pre-birth preparations, immediate newborn care, and post-partum care: 66% vs 80, 72 and 74% respectively (see Table 1).

**Table 1:** Mean participant scores for each skill checklist item for the Non-Breathing Baby scenario

	% Correct Demonstration (*n* = 53)
Pre-birth preparations	
Checked room temperature; turned off fans	80
Washed hands (first of two)	84
Dry cloth placed on mother’s abdomen	78
Prepared the newborn resuscitation area	82
Checked if bag and mask are functional	78
Washed hands (second of two)	78
Wore two pairs of clean gloves	89
Put forceps, cord clamp/ties in easy-to-use order	71
Mean	**80**
Immediate newborn care	
Call out time of birth (hours, minutes, seconds)	73
Drying was started within 5 s after birth	80
Dried the baby thoroughly (wiped the eyes, face, head, front, back, arms and legs)	82
Removed the wet cloth	66
Baby was in direct skin-to-skin contact	68
Covered baby’s body and head with dry cloth	64
Mean	**72**
Neonatal resuscitation	
Called for help	51
Remove first pair of gloves	47
Quickly clamped and cut cord	85
Moved baby to resuscitation area	69
Cover baby quickly during and after transfer	67
Positioned head correctly to open airways	72
Applied face mask firmly over chin, mouth & nose	67
Chest rise within 1 min of birth	60
Squeezed bag to give 30–50 breaths per minute	57
Maintained good chest rise throughout or took steps to improve ventilation	64
Once baby’s breathing well, stopped mechanical ventilation	89
Mean	**66**
Immediate postpartum care	
Returned to skin to skin contact, covered baby	90
Checked for another baby	69
Gave oxytocin to the mother	77
Delivered placenta	53
Counselled mother that baby is ok and on feeding cues	83
Mean	**74**

### Skill retention by cadre

**Fig. 5:**
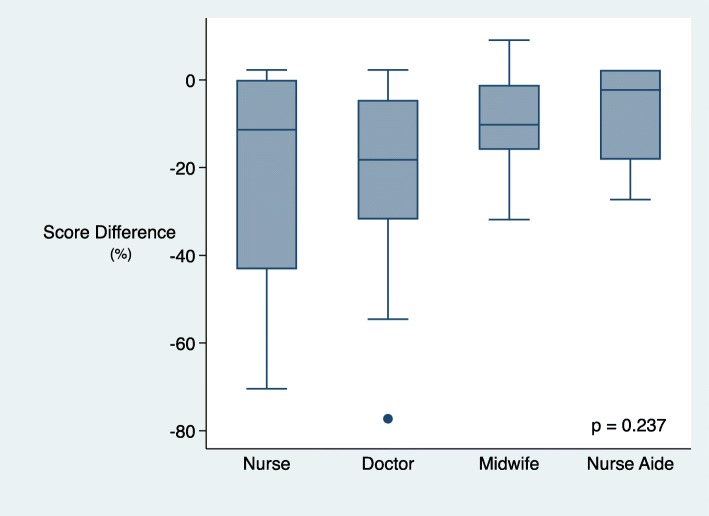
Score difference (post-coaching vs evaluation) in breathing baby scenario by cadre

**Fig. 6:**
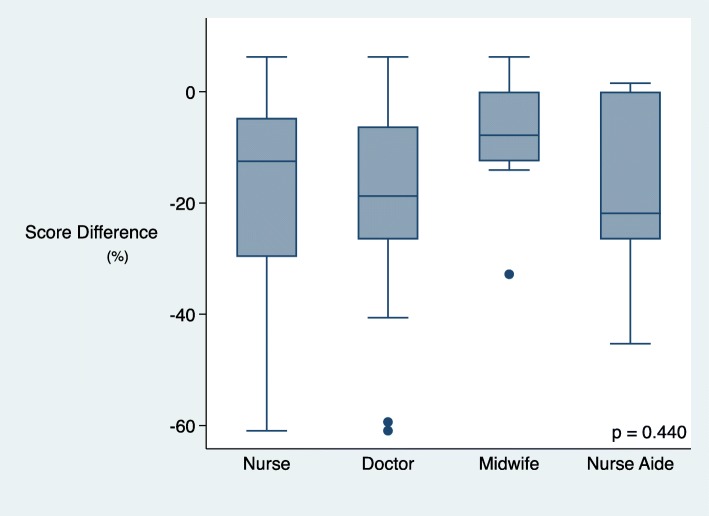
Non-breathing baby scenario score difference (post training vs evaluation) by cadre

When separated by cadre of healthcare worker, nurse aides showed the best skill retention in the breathing baby scenario, and midwives in the non-breathing baby scenario when post-coaching and evaluation scores were compared (Figs. 5 and 6). In the breathing baby scenario, nurse aides had a median score reduction of − 2% (IQR − 18 to 2), midwives − 10% (IQR − 16 to − 1), nurses − 11% (IQR − 43 to 0) doctors − 18% (IQR − 31 to − 4) (*p* = 0.237). In the non-breathing baby scenario, midwives had a median score difference of − 8% (− 13 to 0), nurses − 13% (− 30 to − 5), doctors − 19% (IQR − 27 to − 6) and nurse aides − 22% (IQR − 27 to 0) (*p* = 0.440).

### EENC feedback

Most participants found the content in the EENC program “just right” (83%). 7% found the course “a little too complicated”, 5% “a little simple”, and 5% “too simple”. The mean Likert Scale score response was 3 (SD 0.5) equating to “just right”.

## Discussion

The 2-day EENC coaching intervention delivered by a small team of Solomon Islands Ministry of Health staff in a low-resource Pacific Island setting, increased knowledge by 45%, and skills by 75% (Breathing Baby) and 23% (Non-Breathing baby) respectively. At evaluation after a median of 21 months post-training, skills experienced a modest reduction to 82% (Breathing Baby) and 72% (Non-Breathing Baby). Skills related to pre-birth preparation and immediate post-partum care were better retained than for neonatal resuscitation. Nurse aids and midwives who were engaged in providing routine neonatal care had least reductions in scores at evaluation. Health professionals who did not use the skills routinely had greater reductions in scores at the time of evaluation, but still remained significantly above baseline. The EENC program content was well received by participants.

These findings may have implications for the design and implementation of newborn training in other similar settings. The EENC program uses a practical, coaching methodology with a focus on two practical scenarios using minimal resources, without didactic or excess content over a 2-day training period. Case based learning and clinical simulations with frequent repetition have been shown to be effective educational methods for health care worker training [14]. Short training periods limit time away from clinical posts and are less expensive. This program was implemented through the actions of a small team dedicated to a large geographical area with little opportunities to follow up. More strategies for implementing programs in such settings with limited resources are needed.

Our study found better retention of skills and knowledge in midwives and nurse aides compared to other cadres, reflective of other studies where birth volume and associated frequent clinical practice was associated with better retention [15]. Of concern was the relatively higher drop-off of scores for doctors in both scenarios. Doctor’s skills are often deferred to in resuscitation or for the care of critically ill neonates. For health care workers who infrequently attend deliveries, periodic refreshers and regular practice with portable simulators can maintain skills [16, 17]. Consideration needs to be given to strategies that fit the local context, particularly those that have feasibility in the confines of geography and financial limitations. These could include requirements for continuing education, strengthening local rather than national coordinator roles or providing incentives.

High post-coaching scores in this study demonstrate effectiveness of the training approach in improving knowledge and skills, a result similar to other studies, which range from improvements of between 7 to 89% from baseline [18]. Knowledge and skill improvements are also reflected in national surveys identifying higher rates of adherence to skin-to-skin initiation and duration [19]. The higher Non-Breathing Baby pre-coaching scores compared with Breathing Baby scores are likely due to a boosted score, from this assessment taking place second, with participants incorporating newly learnt skills from the first scenario. Knowledge and skills fall-off occurred in half of 10 studies identified in a systematic review on this theme, with three showing no fall-off and two with a range of retention according to cadres of birth attendants and prior training exposure [18]. Knowledge and skills fall off in the range described in our study has been reported following neonatal training programs [18, 20, 21] and has led to the addition of quality improvement approaches [22] incorporating various strategies such as daily bag and mask skills practice, peer review and weekly skills checklist to improve retention [23]. Whilst establishment of a quality improvement team within 3 months of coaching was recommended with relevant guidance in the EENC modules, they were not utilised at the hospital level in contrast to other countries in the region using EENC where quality improvement implementation occurred up to half the facilities [19, 24]. This was likely due to the small training team in Solomon Islands not being available to facilitate, mentor and support participants from remote geographical locations, with visits occurring not more than once per year due to financial and logistical challenges.

Further analysis of the skills demonstrated showed that resuscitation skills, specifically those related to bag and mask ventilation, were retained less than skills for routine care. This may be due to the lower frequency of resuscitation events in practice, and the greater degree of technical expertise required. An analysis of facilitator and learner perceptions from a neonatal resuscitation program across two low- and middle-income countries found additional training was required to establish resuscitation skills and recommended continued learning and active mentoring to establish this practice [25]. An intervention for Helping Babies Breathe in Nepal, used a neonatal resuscitation protocol which focused on bag and mask ventilation included training, daily skill checks, preparation for resuscitation at every birth, self-evaluation and peer review [23]. Skills and competencies in care of high-risk babies improved following supportive supervision and monitoring in Uganda [26]. Through additional exposure, these additional measures may assist in resuscitation through familiarisation with a typically stressful, low frequency event, of higher complexity.

This study has some limitations. There was no comparison group, and time between coaching and evaluation varied due to logistics and accessibility. Only one participant from each coaching group was assessed at baseline. This reflected a pragmatic approach, where limited resources and time for the coaching were balanced with research needs. Some of the participants in this study had prior neonatal training but none had additional newborn training other than EENC during the study period. Our study used knowledge and simulation to assess healthcare worker skill performance. Ideally, evaluation of training programs would be through measuring impact on neonatal morbidity and mortality, and neurodevelopmental outcomes [10]. However, in practice, these outcomes are difficult to measure due to the sample sizes required, and a research infrastructure that is out of reach of small teams on low budgets. Assessments of knowledge and skills provide indirect information on effectiveness of neonatal resuscitation programs [10] and a systematic review identified four out of five studies with a positive correlation between simulated test scores and clinical behaviour [18]. Other training programs have used similar assessment time points in assessing knowledge and skills [27].

## Conclusion

The EENC coaching program was implemented by a small team and resulted in improved knowledge and skills, especially among those who performed immediate newborn care routinely; however there was a fall-off within 18 months especially among those who did not use the skills routinely. Routine newborn skills were sustained more than resuscitation skills. Complementary strategies are needed to sustain resuscitation skills following coaching over time with novel methods required to reach remote health workers who have infrequent opportunities for resuscitation practice.

## Supplementary information

**Additional file 1.** Written Survey. Written Questionnaire for participant background, experience and training satisfaction scores.

## Data Availability

According to the regulations of the University of Melbourne, the dataset generated and analysed during the study, cannot be made public. The data are however available from the corresponding author (ST) for inspection upon reasonable request.

## Abbreviations

EENC: Early Essential Newborn Care
IQR: Inter Quartile Range
MHMS: Ministry of Health and Medical Services
SIDS: Small Island Developing State
WHO: World Health Organization
