# Supplementary material for: Healthcare worker knowledge and skills following coaching in WHO early essential newborn care program in the Solomon Islands: a prospective multi-site cohort study
Source: BMC Pregnancy Childbirth. 2020 Feb 7;20:84. doi: 10.1186/s12884-020-2739-z (PMC7007662; doi:10.1186/s12884-020-2739-z)
Supplement: Supplementary file 1 — Additional file 1. Written Survey. Written Questionnaire for participant background, experience and training satisfaction scores. [file 12884_2020_2739_MOESM1_ESM.pdf]

WRITTEN SURVEY - CONFIDENTIAL

Location:____ **Date:** ________________________

Participant name: _____________________________________ **Age:** _________________________

1. Your Background

1) What is your role? (please circle)

Nurse Nurse aide Midwife Doctor

2) What are your qualifications? ____________

3) How many years of work experience do you have? ___________________

4) What is your current place of work? (please circle)

NRH Gizo Kirakira Good Samaritan Kilu'ufi

5) How long have you worked at your current place of work? _____ months _____ yrs

6) List the places you have worked in the last 5 years: ____

____

2. Neonatal Experience

1) Have you had EENC training? (please circle) No Yes

2) When did you have you had EENC training? Year _____ Month ______ Location _____________

3) Have you had EENC refresher training before? (please circle) No Yes Year _____ Month ______

4) Have you participated in EENC Health Facility Strengthening? No □ Yes □ Year _______ Month______

5) Have you had any other neonatal training before: No □ Yes □ (circle below and write year)

Integrated Management Childhood Illness ____________

WHO (Blue) Pocketbook ____________

Solomon Island National University Training ____________

Baby Friendly Hospital Initiative ____________

Other___________ year ________

3. EENC training

Was the EENC course too complicated, too simple, or just right? (please circle)

1. Too complicated
2. A little complicated
3. Just right
4. A little simple
5. Too simple
